# Kinesiophobia could affect shoulder function after repair of rotator cuff tears

**DOI:** 10.1186/s12891-022-05679-x

**Published:** 2022-07-26

**Authors:** Huihui Wang, Fangning Hu, Xiaolong Lyu, Honglei Jia, Bomin Wang, Fanxiao Liu, Yongliang Yang

**Affiliations:** Department of Orthopaedics, Shandong Provincial Hospital affiliated to Shandong First Medical University, No.324, Road Jing Wu Wei Qi, Jinan, 250021 Shandong China

**Keywords:** Fear of movement, Rotator cuff injury, Outcome measures, Psychology

## Abstract

**Purpose:**

Kinesiophobia (fear of movement) is a major limiting factor in the return to pre-injury sport level after surgery of rotator cuff tears. The study aims to gain insights into how kinesiophobia affects shoulder pain and function after the repair of full-thickness rotator cuff tears.

**Methods:**

A prospective study was conducted to evaluate patients who underwent rotator cuff repair between January 2019 and December 2019 in our institution. The patients were divided into a trial group with a high kinesiophobia (Tampa Scale for Kinesiophobia [TSK], TSK > 37) and a control group with a low kinesiophobia (TSK ≤ 37). The indicators of interest included the Constant-Murley scores, numerical rating scale (NRS), visual analogue scale (VAS), Oxford Shoulder Score (OSS), and the American shoulder and elbow score (ASES), shoulder function and strength, and range of motion (ROM) at 3 days, 6 weeks, and 12 months after repair of full-thickness rotator cuff tears.

**Results:**

In total, 49 patients who underwent repair of full-thickness rotator cuff tears were enrolled, which was divided into a trial group involving 26 patients (mean TSK 52.54) and a control group involving 23 patients (mean TSK 33.43). There were no statistically significant differences in basic information such as age, gender, and length of stay in the two groups. The preoperative and early postoperative functional scores and the Tampa Scale for Kinesiophobia were statistically significant differences between the two groups. However, long-term postoperative follow-up showed no statistically significant difference in ASES, and Constant-Murley scores, OSS, and VAS scores between the two groups as the kinesiophobia changed from positive to negative.

**Conclusion:**

Degree of kinesiophobia reduced during post-operative rehabilitation of rotator cuff repair patients, but high kinesiophobia is still present in a large portion of the patients after rotator cuff repair. Patients after rotator cuff repair will benefit from early recognition and prevention of kinesiophobia.

## Introduction

A stronger subacromial compression and a higher dynamic eccentric load produced by sport-specific movements usually lead to pain and injury of the rotator cuff tendons. Psychological factors were almost the most influential points on the effection of intervention for shoulder pain. In the last 2 decades, fear of movement took the most of musculoskeletal pain [1]. Kinesiophobia is an extreme form of fear of movement, defined as an excessive, irrational, and debilitating fear of performing a determined movement or activity due to a sense of vulnerability to a painful injury or reinjury. Kinesiophobia is often associated with escape behaviors such as hypervigilance or avoidance [2], which had a negative influence on the range of movement (ROM) in patients with chronic musculoskeletal pain [3], leading to a reduced function of the shoulders. Kinesiophobia is cross-sectionally associated with and longitudinally predicts greater pain intensity, disability, and poor quality of life over time [4]. Numerous studies [2–4] demonstrated that kinesiophobia might be associated with low education, cognitive level, and awareness. Moreover, a high level of kinesiophobia may negatively influence the short-term recovery of these patients, by putting them at higher risk for falling and reduced functionality [4].

In a clinical setting, kinesiophobia is a barrier to physical activity [5]. Consequently, kinesiophobia becomes a targeted outcome in clinical practice [6]. The role that kinesiophobia plays in chronic shoulder pain intensity and disability has been explored [7]. There was a high prevalence of kinesiophobia among patients who underwent total hip arthroplasty, shoulder arthroscopy, spinal surgery, and anterior cruciate ligament reconstruction [6, 8–10]. Pain intensity is directly correlated with the presence of kinesiophobia. Recently, multiple studies [11, 12] have focused on the shoulder pain caused by partial-thickness rotator cuff tears, and indicated that compared with interstitial and articular-sided partial-thickness RCTs, bursal-sided tears can result in more severe shoulder pain. However, the effect of kinesiophobia on postoperative function was not evaluated.

The study aims to gain insights into how kinesiophobia affects shoulder pain and function after the repair of full-thickness rotator cuff tears. The hypothesis is that patients after rotator cuff repair will benefit from early recognition and prevention of kinesiophobia, and kinesiophobia could affect shoulder function after repair of rotator cuff tears.

## Material and methods

This prospective study was approved by the ethics committee of our institution. The data were prospectively collected as part of the standard process, and conducted to evaluate patients who visited the trauma two department of Shandong Provincial Hospital from January 2019 to December 2019. All these patients underwent the operation of full-thickness rotator cuff tear repair. Patients’ characteristics (age, sex, diagnosis, body mass index, etc) were recorded from the patients’ medical files and reviewed by two single researchers.

The inclusion criteria: (1) patients with full-thickness supraspinatus tear of the rotator cuff, who were diagnosed by medical history, physical examination (positive active painful arc test, drop arm test, and weakness in external rotation), imaging examination (ultrasound, MRI or MRA) or diagnostic arthroscopy; (2) aged ≥18 years; (3) no obvious trauma history; e4) unilateral or bilateral; (5) the ability to complete a written survey in Chinese.

Exclusion criteria: (1) the age of patients were less than 18 years; (2) tears involved in teres minor, infraspinatus or and subscapularis; (3) irreparable rotator cuff tears; (4) the presence of other systemic or local diseases leading to shoulder function; (5) history of shoulder surgery or trauma; (6) an inability to understand written and spoken Chinese; and (7) history of psychosis.

### Surgical procedures

Supraspinatus tendon repair was performed in a standardized manner by a mini-open approach. Surgeries were performed with the patient in the beach-chair position and consisted of diagnostic arthroscopy of the glenohumeral joint, a deltoid-splitting approach for tear exposure, an acromioplasty, mobilization of the torn tendons until full coverage of the footprint was achieved, tendon repair by a modified Mason-Allen technique (two to four sets of nonabsorbable sutures, depending on tear size), and repair of the detached part of the deltoid to the acromion through bone tunnels [13]. All patients underwent procedures performed by two experienced orthopaedic surgeons (WBM &YYL).

Postoperatively, the arm was immobilized in a shoulder sling and was helped to exercise a passive range of motion; a detailed plan of the exercise was given to patients. After hospital discharge, patients were supervised and reminded to follow the plan as strictly as possible. We monitored our patients to make sure that they strictly follow our guidance and helped them to over their difficulties during recovery and evaluate their pain and shoulder function. Active-assisted motions were started 6 weeks after surgery, with strengthening exercises initiated 12 weeks after the surgery [13]. A team consisting of doctors and rehabilitation nurses (all trained how to do functional exercise and evaluate the questionnaires in the same way) help the post-operative patients to practice their functional exercise on the day they return to the ward from operative room. A list, explaining when and how to proceed with the exercises with pictures and detailed words would be given to the patients when they discharge from the hospital. A follow-up of at least 12 months by the study team. Brace fixation one-six days after surgery to start fist-clenching exercises, and deltoid isometric exercises. Swing exercises and passive movement of the shoulder joint were started 3 days after surgery. Passive range of motion of the shoulder should be gradually restored four − 5 weeks after surgery. Active shoulder movement from 6 weeks after surgery. Gradual increase in active shoulder range of motion 8 weeks after surgery. Active activity near normal at 12 weeks postoperative. We teach and supervise patients how to fill in the questionnaire and perform rehabilitation exercises in detail and accurately twice (pre and post-operation) to guarantee patients finish the questionnaire in the same way.

### Tampa scale for Kinesiophobia

The Tampa Scale for Kinesiophobia (TSK) is a 17-item scale designed for use in adults to measure fear of movement-evoked pain and injury [14, 15]. Items are scored on a 4-point Likert scale as follows: 1 = strongly disagree; 2 = disagree; 3 = agree; 4 = strongly agree. It contains 13 positively scored items for which endorsing strong agreement is associated with the highest Likert scale score (4), and four reversed scored items, spread out evenly throughout the scale, for which endorsing strong disagreement is associated with the highest score (4) after reverse scoring. Total scores range from 17 to 68 with higher scores indicative of greater fear of movement. The TSK has been shown to have good internal consistency with Cronbach alpha’s ranging from 0.68 to 0.86 [16, 17]. The TSK has demonstrated good test-retest reliability [16]. Patient-reported outcome measures, collected online (WeChat group entitled as “patients number+date of operation”) as part of the standard procedure to monitor their shoulders’ function (the degree of their pain and motion spectrum). These five questionnaires were sent out and answered electronically or on paper. Patients were investigated by the kinesiophobia (Tampa Scale for Kinesiophobia, TSK-17) [17, 18] before their operation, and according to their scores, patients were divided into two groups including a trial group with a high kinesiophobia (TSK > 37), and a control group with a low kinesiophobia (TSK ≤ 37) [19].

#### Outcome of interest

Two groups were asked to fill in the following questionnaires including Numerical Rating Scale (NRS), Visual Analogue Score (VAS), Oxford Shoulder Score (OSS), and the American shoulder and elbow score (ASES) on the spot of preoperatively (T0), postpoerative at T1 (discharge: usually 3 days), 1 week (T2) and follow-ups such as, T3 (6 weeks), and T4 (12 months). We use the NRS and VAS to assess patients’ subjective pain.

### Statistical analysis

We used IBM SPSS Statistics 23 statistical software to analyze the data. Data on parameters such as age, hospitalization days, BMI (Body Mass Index), shoulder function score and Tampa Scale for Kinesiophobia were described using the mean ± standard deviation and t-tests were used for comparison between groups at postoperative 12 months. The skewness and kurtosis, graphical methods, and non-parametric tests was used for normality tests, and the Mann Whitney test was used for nonparametric comparison of continuous variables. Proportional values such as gender, history of tobacco and alcohol and left or right were compared using χ2 -analysis or Fisher’s exact test. The statistical significance level was considered as *p*-value ≤0.05.

## Results

Of all patients with rotator cuff injuries who met the inclusion criteria, 26 were kinesiophobia positive and 23 were kinesiophobia negative. There were no statistically significant differences in age, gender, history of tobacco and alcohol, left or right, hospitalization days and BMI between the groups. All patients underwent surgery as soon as possible after admission and were discharged on the third postoperative day (Table 1). The preoperative rate of agoraphobia was 53% and all patients were without agoraphobia at the final postoperative follow-up.

**Table 1:** The basic information of the included patients

Group	Trial	Control	P
No. of cases	26	23	–
Gender (M/F)	16/10	14/9	0.346
Age (Year, mean ± SD)	54.27 ± 8.84	53.04 ± 8.90	0.631
BMI	24.28 ± 2.90	23.90 ± 2.13	0.610
History of tobacco and alcohol (Y/N)	9/17	6/17	0.528
Left or Right (L/R)	9/17	9/14	0.750
Hospitalization days (d, mean ± SD)	5.88 ± 0.91	5.87 ± 0.87	0.953

*SD* Standard deviation

Preoperatively, we compared the shoulder function scores of the patients and found that the kinesiophobia, OSS, and VAS were significantly higher in the trial group than in the control group (*P* < 0.05). The Constant-Murley score, and ASES were significantly lower in the control groupthan in the trial group (*P* < 0.05) (Table 2).

**Table 2:** Preoperative follow-up results between two groups

Group	Trial	Control	P
Tampa Scale for Kinesiophobia (mean ± SD)	52.54 ± 1.30	33.43 ± 1.67	0.001
OSS (mean ± SD)	51.27 ± 5.68	46.96 ± 4.61	0.001
ASES (mean ± SD)	23.04 ± 1.80	25.96 ± 2.20	0.001
Constant-Murley score (mean ± SD)	19.19 ± 3.45	23.87 ± 3.60	0.001
VAS (mean ± SD)	7.42 ± 0.58	6.70 ± 0.63	0.001

*ASES* American shoulder and elbow score, *SD* Standard deviation, *OSS* Oxford Shoulder Score, *VAS* Visual analogue scale

We followed each patient for 12 months after the operation. There were significant differences between the two groups in the first 6 weeks on the kinesiophobia and shoulder function scores. At the 6 weeks postoperatively, there was no significant difference in the kinesiophobia and shoulder function scores between the trial group and control group (*P* > 0.05) (Table 3). From the sixth week after surgery until the end of follow-up, there were no significant differences in the kinesiophobia and shoulder function scores between the two groups (*P* > 0.05) (Table 4).

**Table 3:** Postoperative follow-up results (6 weeks) between two groups

Group	Trial	Control	P
Tampa Scale for Kinesiophobia (mean ± SD)	32.96 ± 1.48	28.17 ± 1.03	0.06
OSS (mean ± SD)	34.69 ± 1.91	34.09 ± 1.28	0.21
ASES (mean ± SD)	45.54 ± 3.68	45.52 ± 3.80	0.99
Constant-Murley score (mean ± SD)	45.42 ± 3.82	45.83 ± 3.70	0.71
VAS (mean ± SD)	5.04 ± 0.96	5.26 ± 0.81	0.39

*ASES* American shoulder and elbow score, *SD* Standard deviation, *OSS* Oxford Shoulder Score, *VAS* Visual analogue scale

**Table 4:** Postoperative follow-up results (12 months) between two groups

Group	Trial	Control	P
Tampa Scale for Kinesiophobia (mean ± SD)	27.42 ± 1.47	26.87 ± 1.18	0.16
OSS (mean ± SD)	13.85 ± 0.92	13.39 ± 1.12	0.13
ASES (mean ± SD)	88.96 ± 2.99	90.26 ± 3.05	0.14
Constant-Murley score (mean ± SD)	93.54 ± 1.30	94.04 ± 1.64	0.24
VAS (mean ± SD)	1.35 ± 0.49	1.35 ± 0.49	0.99

*ASES* American shoulder and elbow score, *SD* Standard deviation, *OSS* Oxford Shoulder Score, *VAS* Visual analogue scale

## Discussion

The prevalence of kinesiophobia decreases during rotator cuff repair patients’ postoperative rehabilitation, but high kinesiophobia is still present in a large portion of the patients after rotator cuff repair. The present study showed that significant differences in the first 6 weeks between the two groups, confirming our hypothesis that kinesiophobia could affect shoulder function after repair of rotator cuff tears.

Higher pain intensity is associated with fear-avoidance behavior [20]. Fear of pain always leads to more severe disability in both chronic and acute pain conditions [21]. Movements fear can cause a negative vicious cycle and greater pain level (acute, subacute, and chronic), progression of disability over time, emotional distress [1], and lower quality of life [22–26]. Kinesiophobia group in our study needs more guidance and supervision to help them to overcome, who have a high level of NRS and VAS, and a low level of, OSS, and ASES in the 8 weeks post-operation. However, in the long run, that is 10 weeks- 12 months, the gap between the high and control group decrease.

Kinesiophobia is well known as a negative factor of rehabilitation adherence in different chronic pain conditions [27, 28], predicted more chronic musculoskeletal pain and disability over time [29]. Furthermore, it can be made good use of as a modifiable factor, and earlier achievement of pain relief and functional recovery are possible [30]. We clinicians should raise the awareness of the presence of kinesiophobia and give more specific interventions and education, cognitive-behavioural activities, exercise therapy if necessary, which could produce huge benefits in individuals with chronic musculoskeletal pain [20, 31]. Maladaptive behaviors mean a barrier to practice exercise, which hampers recovery [32, 33]. Kinesiophobia has some association with shoulder function [34]. Greater levels of kinesiophobia were associated with greater levels of chronic shoulder pain intensity and disability [35]. A broad list of biopsychosocial factors is associated with the transition and perpetuation of chronic shoulder pain [36]. In all, kinesiophobia is considered a moderator of treatment response among individuals with musculoskeletal pain [37, 38].

Recognize the positive kinesiophobia scores patients and give them early intervention like health education, nursing, and rehabilitation to help them overcome their fear about motion can endure the effect of the operation, to erase their pain as much as possible, and gain more usual shoulder’s function. It is a bottleneck for patients with the repair of rotator cuff tears at this stage of the range from 1 week to 3 months especially from 4 weeks to 3 months. Exercise itself can help overcome fear [39]. In addition, physical activity can also improve the exercise self-efficacy of individuals with restricted activities [39]. Education can eliminate patients’ excessive fear of the perception of physical vulnerability, pain neurophysiology, movement disorders, also it can improve exercise benefits and increase the population’s physical activity [1]. When formulating a rehabilitation plan and conducting health education and guidance for patients after rotator cuff injuries repair, it is necessary to fully consider the significant differences in the exercise capacity and tolerance of different patients, and according to different ages, occupations, and needs, patients have different needs for shoulder function recovery. Therefore, On the basis of needs, considering TSK scores, it is recommended that the attention and progress of the rehabilitation program can be adjusted appropriately with everyone’s timely follow-up. It varies from person to person. Each patient is a different individual, case managers might be a good choice [40]. If the compliance is very unsatisfactory and the rehabilitation progress is seriously delayed, the best exercise opportunity may be missed, resulting in adhesions. Possible injuries and adverse events should be assessed during rotator cuff injury patients’ postoperative rehabilitation. Compared with the rehabilitation of other diseases, the plan detail is clear and specific. The main obstacles to rehabilitation are pain and fear of movement. Serious adverse medical events barely occur. Patients can benefit from effective analgesia and rehabilitation guidance.

This study only conducted intervention studies on a certain type of single surgical patients, the sample size is limited, and the continuous follow-up only lasts 12 months after surgery, which has certain limitations. The influence of patient psychiatric factors on the presence of relevant studies was not taken into account. Many studies [41, 42] focused on kinesiophobia and the involvement of the brain. With the assistance of the functional magnetic resonance imaging, one study [41] attempted to explore the relationship between five clinically scores and brain activation forms in patients with apprehension caused by anterior shoulder instability, which demonstrated the strongest correlation of shoulder apprehension and brain level alterations, whereas pain VAS and WOSI suggested an intermediately strong association recruiting fewer brain networks. Increasing evidence supported the use of a global multimodal strategy including proprioception, mirror therapy, cognitive behavioural methods, surgical stabilization techniques, and traditional physical therapy to minimize persistent micromotion, which may be beneficial for the recovery of brain function [42]. The effect between tear size and postoperative functional outcome needs further study. In the future, the sample size can be expanded, and other types of rotator cuff injuries can be expanded. The surgical procedures can be increased to a trans-shoulder replacement, shoulder arthroscopy, etc., and even the identification and intervention of kinesiophobia can be applied to conservative treatment patients to explore the period of treatment. The impact of the rehabilitation progress and extent of patients with rotator cuff injury. Extend the follow-up time, and compare conservative treatment and surgical treatment to the social and health economic significance of the current social division of labor and aging gradually, in order to choose more suitable treatment and rehabilitation programs for patients.

## Conclusion

Degree of kinesiophobia reduced during post-operative rehabilitation of rotator cuff repair patients, but high kinesiophobia is still present in a large portion of the patients after rotator cuff repair. Patients after rotator cuff repair will benefit from early recognition and prevention of kinesiophobia.

## Data Availability

All data generated or analysed during this study are included in this published article.

## Abbreviations

ASES: American shoulder and elbow score
NRS: Numerical rating scale
OSS: Oxford Shoulder Score
ROM: Range of motion
TSK: Tampa Scale for Kinesiophobia
VAS: Visual analogue scal
